# ASO Author Reflection: Demographic Conditions are the Major Determinants for Functional Outcome and Quality of Life in Lower Extremity Soft Tissue Sarcoma Patients

**DOI:** 10.1245/s10434-021-09781-7

**Published:** 2021-03-02

**Authors:** Gilber Kask, Ian Barner-Rasmussen

**Affiliations:** 1 Department of Plastic Surgery, Helsinki University Hospital and University of Helsinki, Helsinki, Finland; 2 Unit of Musculoskeletal Surgery, Department of Orthopaedics and Traumatology, Tampere University Hospital, Tampere, Finland

## Past

Most soft tissue sarcomas (STS) are located in the lower extremity. A number of previous studies of functional outcome after treatment of lower extremity STS have been published,^1^ but only a few on long-term health-related quality of life (HRQL).^2^,^3^ The literature is heterogeneous as many reports include both upper and lower extremity STS patients, bone tumors, and patients treated with amputation. Most studies have investigated the effect of disease- and treatment-related factors on functional outcome and HRQL. Few studies have focused on patient-related factors.^3^,^4^

## Present

We performed a cross-sectional study in a tertiary referral center using the TESS, QLQ-C30, and 15D measures based on 141 patients who had undergone limb salvage surgery for lower extremity STS.^5^ Mean functional outcome and HRQL were generally good and comparable to previous studies. Treatment-related factors, such as need for reconstructions and radiotherapy, had an impact, but patient-related factors (age and BMI) were the major determinants of both functional outcome and HRQL in the long term.

## Future

Based on our study, patient-related factors have a greater effect on functional outcome and HRQL compared with tumor- and treatment-related factors. Patient demographics should thus be taken into account when reporting functional outcome and HRQL. Additionally, sarcoma studies often include small study samples, increasing the risk of demographical bias. In functional and HRQL outcome studies, other factors possibly influencing functional or HRQL outcome are generally not reported or included in analysis. A deeper understanding of how other factors, such as disease, trauma, or socio-economic circumstances, additionally affect functional and HRQL outcome in extremity sarcoma patients would be valuable. This would help us to better understand STS as a disease and the effect of treatment on functional outcome and HRQL.
